# QTL Mapping of Palmitic Acid Content Using Specific-Locus Amplified Fragment Sequencing (SLAF-Seq) Genotyping in Soybeans (*Glycine max* L.)

**DOI:** 10.3390/ijms231911273

**Published:** 2022-09-24

**Authors:** Yongguo Xue, Huawei Gao, Xinlei Liu, Xiaofei Tang, Dan Cao, Xiaoyan Luan, Lin Zhao, Lijuan Qiu

**Affiliations:** 1 Institute of Soybean Research, Heilongjiang Provincial Academy of Agricultural Sciences, Harbin 150086, China; 2 Key Laboratory of Soybean Biology of Ministry of Education China, Northeast Agricultural University, Harbin 150030, China; 3 National Key Facility for Crop Gene Resources and Genetic Improvemen, Institute of Crop Sciences, Chinese Academy of Agricultural Sciences, Beijing 100081, China

**Keywords:** soybean, SLAF-seq, palmitic acid content, QTL, candidate gene

## Abstract

Soybeans are essential crops that supply protein and oil. The composition and contents of soybean fatty acids are relevant to human health and have a significant relationship with soybean oil processing and applications. Identifying quantitative trait locus (QTL) genes related to palmitic acid could facilitate the development of a range of nutritive soybean cultivars using molecular marker-assisted selection. In this study, we used a cultivar with higher palmitic acid content, ‘Dongnong42’, and a lower palmitic acid content cultivar, ‘Hobbit’, to establish F_2:6_ recombinant inbred lines. A high-density genetic map containing 9980 SLAF markers was constructed and distributed across 20 soybean chromosomes. The genetic map contained a total genetic distance of 2602.58 cM and an average genetic distance of 0.39 cM between adjacent markers. Two QTLs related to palmitic acid content were mapped using inclusive composite interval mapping, explaining 4.2–10.1% of the phenotypic variance in three different years and environments, including the QTL included in seed palmitic 7-3, which was validated by developing SSR markers. Based on the SNP/Indel and significant differential expression analyses of Dongnong42 and Hobbit, two genes, *Glyma.15g119700* and *Glyma.15g119800*, were selected as candidate genes. The high-density genetic map, QTLs, and molecular markers will be helpful for the map-based cloning of palmitic acid content genes. These could be used to accelerate breeding for high nutritive value cultivars via molecular marker-assisted breeding.

## 1. Introduction

Soybeans are one of the world’s most essential crops for oil and vegetable protein (http://soystats.com/, accessed on 1 January 2022). Soybean oil contains essential unsaturated fatty acids, such as linoleic acid and linolenic acid, which have essential nutritional and health values for humans [1,2,3]. There are five main types of soybean fatty acids: the saturated fatty acids, palmitic acid (PA) and stearic acid (SA), and the unsaturated fatty acids, oleic acid (OA), linoleic acid (LA), and linolenic acid (LNA). The mean PA, SA, OA, LA, and LNA contents are 12.2, 3.8, 21.5, 54.2, and 8.3%, respectively [4]. Different fatty acid compositions can determine the specific practical uses of soybean oil [5]. Excessive intake of saturated fatty acids harms the human body and can lead to diseases affecting the cardiovascular system and prostate [6]. Increasing the content of the unsaturated fatty acids, oleic and linoleic acid, can reduce the content of palmitic acid and linolenic acid, and ensure that the fatty acid composition ratio can play an important role in nutritional balance, improve the performance of soybean oil, and prolong its shelf life [7]. On the other hand, soybean oil with increased saturated fatty acids and monounsaturated fatty acid content is more suitable for industrial oil processing and utilization [8].

Palmitic acid (16:0) is one of two primary saturated fatty acids in soybeans, accounting for about 11% of soybean oil [9]. Increased palmitic acid content can improve the stability of soybean oil, whereas lower palmitic acid content can reduce the risk of cardiovascular diseases in humans [10]. Traditional breeding and recent advances in genetic engineering have been used to obtain soybean cultivars with low polyunsaturated fatty acid content and improved oxidative stability without chemical hydrogenation [11,12]. Modification of genes related to palmitic acid content has been reported to develop a suitable germplasm with palmitic acid content (>4 to <40%) that can meet health requirements [10]. In recent years, near-infrared spectroscopy has been used to detect fatty acid components in seeds without damage, especially in agriculture, industrial production, and scientific research [13,14,15,16]. To obtain a more reasonable fatty acid composition of soybean oil for industrial use, and due to greater consumer awareness of health issues, QTL mapping, gene cloning, and identifying molecular markers for palmitic acid content have been used to develop new soybean cultivars with desirable fatty acid compositions through molecular marker-assisted breeding of soybeans.

However, variation in the palmitic acid content of soybeans is influenced by a complex network of genetic and environmental factors [3,17,18]. Palmitic acid slightly affects yield-related phenotypes [19]. The current population size and stability used for palmitic acid QTL mapping are small, and the marker density of the constructed genetic linkage map needs to be increased. In previous studies, only 37 palmitic acid-related QTLs of linkage disequilibrium (LD) and 47 QTLs of a genome-wide association study (GWAS) were mapped (https://soybase.org/, accessed on 1 August 2022). Five genes affecting palmitic acid content have been identified, *fap1*, *fap3*, *fapx*, *sop1*, and *fapnc*, but their allelic relationship is not entirely known. There is molecular evidence that *fapnc* and *fap3* are located at the same locus [10,19]. A disrupted splicing mutation in an allele gene of *fap1*, *3-ketoacyl-ACP* synthase enzyme III (*GmKASIIIA*, *Glyma09g41380*), has been associated with the reduced palmitic acid phenotype [20]. *Fap3* is an allele of the *FATB1a* gene, which codes for a 16:0-acyl carrier protein (ACP) thioesterase enzyme [21]. *Fapnc* represents the second allele of *GmFATB1A*, and its deletion leads to the low palmitic acid phenotype [22]. *Fap3* has a non-synonymous substitution, which negatively affects the *FATB1a* function. In soybeans, seed palmitic acid content is influenced by the expression of the *FAD2-1* and *FatB* genes [22,23]. These genes are useful for MAS in the breeding program to alter the composition of soybean fatty acids. Numerous QTLs related to fatty acid components have been identified. However, the significant differences between genetic backgrounds and environments, poor repetition and stability of QTLs, and inconvenient integration remain problems in our research.

In addition to palmitic acid, overall fatty acid content is a quantitative trait that depends on the combined effects of several major and minor genes [5,17,24]. QTL mapping is an effective method used to reveal the genetic basis of fatty acid formation and, to date, many QTLs for fatty acid content have been detected. However, due to the relatively low density of genetic maps, these QTLs span a considerable genomic region. The relatively low accuracy of QTL mapping using these maps has limited, not only the identification of fatty acid biosynthesis and regulatory networks, but also the application of these QTLs in soybean marker-assisted selection (MAS) breeding efforts [24].

With the publication of the soybean reference genome and continuous development of molecular marker technology, single nucleotide polymorphisms (SNPs) have become a critical component of genetic map construction, map-based cloning, and genome-wide association analysis, and can be used to locate important soybean pathways for complex quantitative traits [25,26]. Researchers have recently used SLAF-seq, the Soybean SNP BeadChip, SNP genotyping and bin map, and other methods to identify genotypes and construct genetic linkage maps. The number of genetic map markers range from 2086 to 8691, and the total genetic lengths are 1478.86–3780.98 cM. The average map distance is 0.4–1.3 cM, and significant progress has been made in important traits of QTL mapping, such as yield [27,28,29,30,31,32,33], quality [24,28,34,35,36], and stress tolerance [37,38,39,40,41]. Specific-locus amplified fragment sequencing (SLAF-seq) is a rapid and large-scale SNP genotyping method based on next-generation sequencing (NGS) technology [42,43], with effective reads and high throughput. It is widely used in genetic map construction, quantitative trait locus (QTL) mapping, and other fields of population genetic research related to molecular breeding [37]. Compared with the difficulty and high costs of chemical detection, molecular marker-assisted breeding is a simple and reasonable approach to improving the palmitic acid content of soybean cultivars [24]. The continuous improvement of soybean genetic mapping based on molecular markers has dramatically improved the speed and accuracy of QTL mapping for many critical agronomic traits and is a powerful method for genetic map construction, QTL mapping, and molecular marker-assisted selection.

In this study, QTL and linkage markers associated with palmitic acid components were obtained and finely mapped based on the phenotypes over three years, and a soybean high-density genetic linkage map was constructed by SLAF-seq technology. The genome and transcriptome were combined to obtain the candidate genes. The high-density genetic map from our research could further explore more effective and novel QTLs in soybeans and identify candidate genes and associated markers that could contribute to the gene cloning, functional verification, and improvement of palmitic acid composition.

## 2. Results

### 2.1. Phenotypic Evaluation

**Figure 1:**
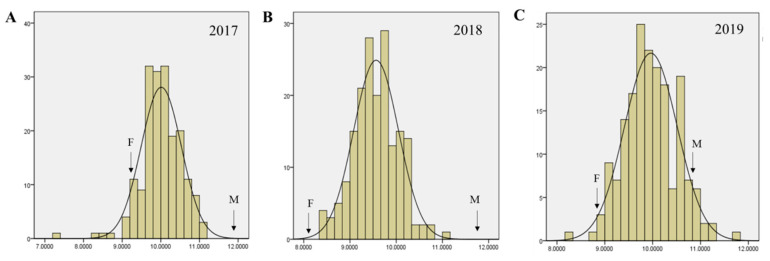
The frequency distribution of palmitic acid content (*x*-axis, %) of the RIL population and parents in (**A**) 2017, (**B**) 2018, (**C**) and 2019. F: female parent (Dongnong42, DN42); M: male parent (Hobbit).

The palmitic acid content of the female parent, Dongnong42, (DN42, Heilongjiang province of China, official registration number: ZDD22643) was significantly higher than that of the male parent, Hobbit, (OH, USA, Chinese official registration number: WDD00573). Over three years, the palmitic acid content of the offspring conformed to a normal distribution, and palmitic acid content within the population varied widely (Figure 1, Table 1). The phenotypes of palmitic acid over the three years—2017 Sanya, 2018 Harbin, and 2019 Suihua—were strongly correlated (Table S1).

### 2.2. SLAF Library Construction and Genotyping of the RIL Population

**Figure 2:**
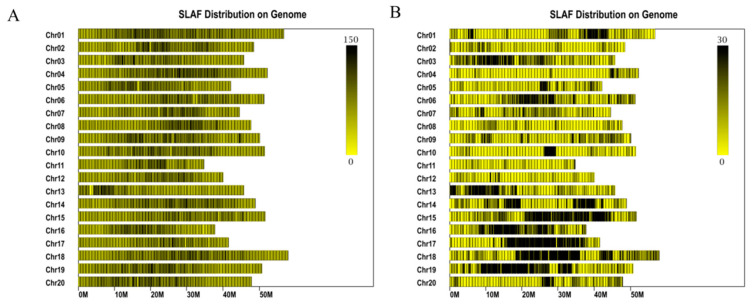
Distribution of SLAF and polymorphic SLAF markers in the genome. (**A**) The picture shows the distribution of SLAF labels. (**B**) Distribution of polymorphic SLAF labels. The abscissa is the length of the chromosome. Each yellow band represents a chromosome. The genome is divided according to the size of 1 M. The darker areas in the figure are the areas where the SLAF tags are concentrated.

Before library construction, sequencing, and genotyping of SLAF-seq, the enzyme combination, *Rsa*I and *Hae*III, was used to digest the genomic DNA of the two parents and the 181 RIL_5_ population. The sequencing depths of ‘DN42’ and ‘Hobbit’ were 120.52× and 117.77×, and the average sequencing depth of the offspring was 22.06×. The enzyme digestion efficiency was 86.62%. A total of 460,888 sequencing reads (Figure 2A), of which 73,579 were polymorphic (Figure 2B), were compared with the soybean genome (http://phytozome.jgi.doe.gov/pz/portal.html, accessed on 1 January 2022). The sequencing Q30 was 84.43%, and the GC content was 40.11%. The pair-end alignment efficiency was 77.96%. A total of 460,888 SLAF markers were obtained, 52,005 markers were successfully encoded, and 48,082 polymorphic markers of the aabb type were selected as practical markers for the population. The effective polymorphisms of the genetic map were 10.43% (Figure S1).

### 2.3. Construction of the Soybean Genetic Map

**Figure 3:**
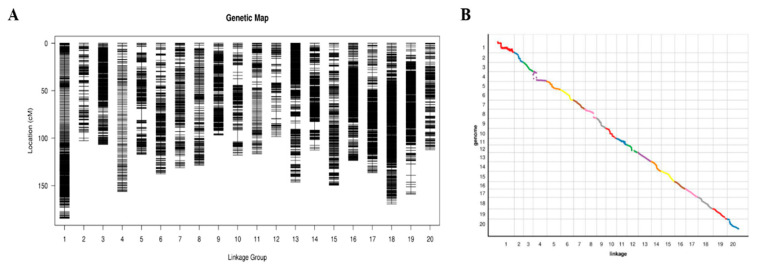
High density genetic map and collinearity analysis of the genetic map and genome. Distribution of markers on 20 chromosomes. (**A**) Black bars on each chromosome represent mapped SLAF-seq markers. The chromosome number is shown on the *x*-axis, and genetic distance (cM) is shown on the *y*-axis. (**B**) The *x*-axis represents the genetic distance of each linkage group. The *y*-axis represents the physical length of each linkage group, with the collinearity of genomic markers and genetic maps represented in scatter. Different colors represent different chromosomes or linkage groups.

A total of 9980 out of 48,082 SLAF markers were used to map out 20 chromosomes. The high-density genetic map had a total genetic distance of 2602.58 cM and an average genetic distance between markers of 0.39 cM, respectively (Figure 3A, Table 2). The collinearity analysis of the position and genetic map of the SLAF markers on the reference genome evaluated the quality of the high-density genetic map (Figure 3B). The average Spearman coefficient was 94.8%, showing the reliable consistency of SLAF markers and the soybean genome (Table 2, Figure 2, Table S2).

### 2.4. QTL Mapping of the RIL Population

The ICIM method was used to map the QTL of palmitic acid content in three environments. Two QTL loci related to palmitic acid were co-located (Table 3) on chromosomes 10 and 15, including three loci, explaining 4.2–10.1% of the phenotypic variance for palmitic acid across environments (Table 3). In Sanya, 2017, the qPA10-1 was mapped between two markers, Marker7632755 and Marker7723217. The odds and the additive effect logarithm were 2.3 and 4.2%, respectively (Figure S2). From 2018 to 2019, we simultaneously located two QTLs, qPA15-1 and qPA15-2, between Marker5381863 and Marker5394023 on chromosome 15, explaining 10.066 and 9.526% of the phenotypic variance, respectively. The logarithms of odds were 6.528 and 3.999, and the additive effects were 0.259 and 0.261, respectively (Figures S3 and S4), which showed these QTLs were associated with a high palmitic acid content.

### 2.5. Verification of Candidate Region and Screening of Linkage Markers

**Figure 4:**
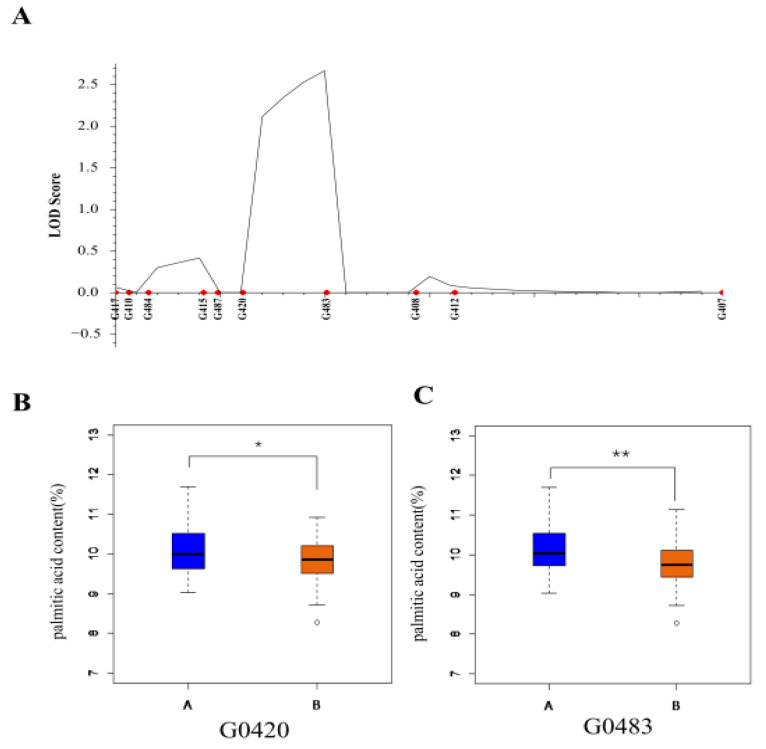
Validation of candidate region and analysis of linkage markers. (**A**) Ten polymorphic markers related to the candidate region with SLAF-seq used to verify the region and screen a QTL region between G420 and G483 markers. (**B**) The lines of the RIL population were divided into two groups based on G420 genotypes. The palmitic acid content of the two groups was significantly different. Student’s *t*-test, * *p* < 0.05. (**C**) The lines of the RIL population were divided into two groups based on G483 genotypes. The palmitic acid content of the two groups was significantly different. Student’s t-test, ** *p* < 0.01. The “A” and “B” represent the genotypes of DN42 and Hobbit, respectively.

Based on the QTL mapping results of SLAF-seq, we screened 10 polymorphic markers from 27 SSR markers (Table S3) related to the candidate region with SLAF-seq to verify the region and screen associated markers. The region between two markers, G420 and G483, was confirmed as the candidate region, and the genotypes of two markers, G420 and G483, were significantly correlated with palmitic acid content (Figure 4).

### 2.6. Analysis of Genome and Transcriptome

After Illumina sequencing and alignment with the reference genome, Glycine max Wm82.a2.v1, 48.84 G of raw data were generated by re-sequencing, of which the raw data yields of DN42 and Hobbit were 25.48 and 23.36 G, respectively, and the filtered clean data were 25.2 and 23.1 G, respectively. The error rate (%) was 0.03, Q30 values (percentage of bases with a Phred value greater than 30 in the total bases) were 94.17 and 94.23%, respectively, and GC contents were 37.5 and 36.99%, respectively. Based on Wm82.a2.v1, seven candidate protein-encoding genes (*Glyma.15g119200–Glyma.15g119800*) were present in qPA15-1 and qPA15-2. The annotations of candidate genes were all bifunctional inhibitors/lipid-transfer protein/seed storage 2 S albumin superfamily protein, whereas *Glyma.15g119300* was not present in Glycine max Wm82.a4.v1 (https://www.soybase.org/GlycineBlastPages/blast_descriptions.php), which may have been caused by genome splicing errors. There were 492 SNPs and 124 Indels in DN42 and Hobbit, and most variation sites existed in the intergenic, upstream, and downstream regions (Table 4). Except for *Glyma.15g119600*, 13 SNPs and 2 Indels were screened based on the coding region between the two parents, which included 12 nonsynonymous SNV, 1 synonymous SNV, 1 non-frameshift deletion, 1 non-frameshift insertion.

**Figure 5:**
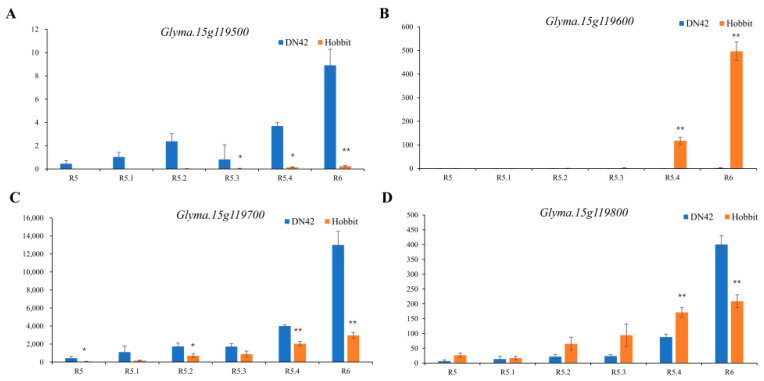
The relative expression (*y*-axis) of the four genes in the candidate region between the growth period of R5–R6 (*x*-axis) in RNA-seq. (**A**) Expression level of *Glyma.15g119500* at the seed development R5–R6 stage. (**B**) Expression level of *Glyma.15g119600* at the seed developing R5–R6 stage. (**C**) Expression level of *Glyma.15g119700* at the seed development R5–R6 stage. (**D**) Expression level of *Glyma.15g119800* at the seed development R5–R6 stage. Bars mean SD. *, *p* < 0.05; **, *p* < 0.01.

To further select candidate genes, the developmental seeds of the six stages (R5–R6) of DN42 and Hobbit were used for seed transcriptome analysis. After the Q-PCR accurately quantified the effective concentrations, the libraries were sequenced on the Illumina platform. After transcriptome analysis, a total of 289.52 G of clean data was obtained; the clean data of each sample reached 5.48 G and the percentage of Q30 exceeded 92.31%. The efficiencies of clean reads of each sample sequence with the reference genome ranged from 91.11 to 95.19%. Based on the transcriptome, we found that three genes, *Glyma.15g119200, Glyma.15g119300, and Glyma.15g119400*, were not expressed in the two parents, and the expression levels of *Glyma.15g119500* in Hobbit and *Glyma.15g119600* in DN42 were deficient. The expression level of *Glyma.15g119700* in DN42 was significantly higher than that of Hobbit at the six periods from R5 to R6. From R5 to R5.4, the expression level of *Glyma.15g119800* in Hobbit was higher than that of DN42, but the difference was not significant in the early stages, and became significant at R5.4. On the contrary, the expression level in Hobbit was significantly lower than that of DN42 at R6 (Figure 5).There was a high similarity in the coding sequence among four genes: *Glyma.15g119500, Glyma.15g119600, Glyma.15g119700, and Glyma.15g119800* (Figure S5).

## 3. Discussion

### 3.1. Construction of the High-Density Genetic Map

Previous studies have shown that relatively low-density genetic maps are not sufficient for the quantitative analysis of trait genes controlled by multiple genes, especially genes with low effects and high susceptibility to the environment [3,44]. According to our statistics, since 2010, about 40 genetic maps have been developed from soybean research on the yield, quality, and resistance to biotic or abiotic stresses (references not shown), with 2086–8691 markers of genetic populations, and total lengths of 1478.86–3780.98 cM. Only six of these maps had higher densities, exceeding 6000 markers [18,37,38,40,45].

In this study, we exploited 181 RIL5 populations to develop 460,888 SLAF markers, and the effective parental polymorphism was 10.43%. The polymorphism SLAF tag of populations was 73,579, located in 20 linkage groups. Finally, 9980 SLAF markers were constructed on the genetic map, with the total genetic distance and average map distances being 2602.58 cM and 0.39 cM, respectively. Compared with previous studies, the marker density of the genetic map in this study was still very high. SoySNP50K was used to detect two populations to construct two genetic maps with 21,478 and 11,922 markers, and, with the updating of the Glyma1.01 build based on two high-resolution linkage maps, the genomic positions of the commonly used markers in the BARCSOYSSR_1.0 database and SoySNP50K BeadChip were updated, based on the Wm82.a2.v1 assembly [46]. The high-density genetic map from this study could facilitate the identification of genes or QTL controlling yield, seed quality, and tolerance of biotic or abiotic stress, as well as other factors.

### 3.2. QTL Mapping of Palmitic Acid Content

The fatty acid content of soybean has high variability, and the palmitic acid content of inbred lines is significantly higher than that of landraces, and is greatly affected by the environment [4,18]. Previous studies have shown that reduced palmitic acid content may lead to increased oil content [47]. Currently, numerous QTLs related to palmitic acid have been identified, with 39 QTLs identified on 15 of 20 chromosomes and 47 reported SNPs (https://soybase.org/, accessed on 1 August 2022). In recent years, with the continuous development of genotyping techniques, a large number of palmitic acid-related QTLs [24,48,49,50], SNP loci [44,51,52], and associated markers [48,50,53,54,55,56] based on palmitic acid content-related research have been gradually developed and partially applied to molecular breeding.

Based on our genetic map, we located a QTL on chromosome 10, explaining 4.151% of the phenotypic variance in 2017 Sanya. This QTL was not repeatedly located in the following two years and may be an environment-specific QTL. Across two environments, *qPA15-1* and *qPA15-2* on chromosome 15 were repeatedly identified, with LOD values of 6.5 and 4.0, and explained 10.1 and 9.5% of the phenotypic variance, respectively. This QTL may also be affected by the environment, which may be why it was not detected in 2017 Sanya. Based on the QTL mapping region of SLAF-seq, we screened two markers based on 27 SSR markers, G420 (Gm15:9,358,076) and G483 (Gm15:9,466,527), which were associated with palmitic acid based on the RIL population derived from DN42 × Hobbit. This mapping region of chromosome 15 has been reported previously [53], shown as Seed palmitic 7-3 (https://www.soybase.org/) with two associated markers, Sat_273 (Gm15:9,396,584) and BARC–054023–12243 (Gm15:14,779,070). The QTL Seed palmitic 7-3 was too large to complete fine mapping. However, the mapping region of this research was small, at only 168.911 kp, and seven genes refer to Glycine max Wm82.a2.v1 (https://phytozome-next.jgi.doe.gov/info/Gmax_Wm82_a2_v1).

### 3.3. Analysis of Candidate Genes

High palmitic acid levels could improve the oxidative stability of soybean oil, and reducing palmitic acid in oil (16:0) from 11 to <4% makes it possible to achieve a low content of saturated fatty acids, which is desirable for cardiovascular health [11]. In soybeans, significant efforts have been made to identify and characterize mutant alleles related to palmitic acid content to improve oil composition [57,58]. For example, mutations at *GmFATB (Glyma05g08060 (Wm82.a1.v1) and Glyma.05g012300 (Wm82.a2.v1))* present low palmitic acid (as low as 5.6%) [10]. New alleles of *GmFATB1A (Glyma.05g012300)* elongate FATB with an acyl-ATP thioesterase, which could reduce palmitic acid levels [59]. *Glyma.14g27990 (Glyma.14g121400)*, coding Stearoyl-ACP desaturase isoform C (*SACPD-C*), could influence palmitic and stearic acid [60]. A beta-ketoacyl-[acyl-carrier-protein] synthase III gene (*KAS* *III* */* *Glyma09g41380,* *Glyma.* *09g* *277400*) was highly correlated with the reduced palmitic acid phenotype due to *fap1* [20]. Candidate genes of Abscisic Acid Insensitive 3 (*ABI3, Glyma18g38490, Glyma.18g176100*) were significantly associated with palmitic acid concentration, explaining 5.42% of phenotypic variation [61]. However, the small number of genes that have been published are insufficient for the identification and breeding of ideal soybean varieties of specific palmitic acid content. We analyzed the expression levels of the four published palmitic acid-related genes between parents, DN42 and Hobbit. Except for *Glyma.09g277400* in the R6 stage and *Glyma.18g176100* in the R5 stage, the expression levels of the four genes in the other seed development stages were not significantly different(Figure S6). In the candidate region of our study, *Glyma.15g119300* was not present in Glycine max Wm82.a4.v1, which may have been caused by genome splicing errors. Twelve nonsynonymous SNVs existed in the coding regions of the six genes. Interestingly, the identical gene annotation of seven genes was consistent: bifunctional inhibitors/lipid-transfer protein/seed storage 2 S albumin superfamily protein. After transcriptome analysis, we found that *Glyma.15g119200, Glyma.15g119300*, and *Glyma.15g119400* were not expressed in the two parents; and the expression level of *Glyma.15g119500* in Hobbit and *Glyma.15g119600* in DN42 were deficient. Furthermore, through the genomic information of DN42 and Hobbit, and we found that there were two and five non-synonymous mutations in *Glyma.15g119700* and *Glyma.15g119800*, respectively, so we reasoned that these two genes were the candidate genes for the palmitic acid content. Four genes with high similarity in coding sequence, *Glyma.15g119500, Glyma.15g119600, Glyma.15g119700, and Glyma.15g119800* (Figure S5), may act as a repeating sequence. However, further research is required to confirm candidate genes and mechanisms. Identifying QTLs, linkage markers, and candidate genes may help improve soybean oil composition traits to further enhance soybean oil quality.

## 4. Materials and Methods

### 4.1. Plant Material and Phenotyping

Soybean RIL population lines and their parents, Dongnong42 (DN42, Heilongjiang province of China, official registration number: ZDD22643) and Hobbit (OH, USA, Chinese official registration number: WDD00573), were grown in three different environments (Evr.1–Evr.3), including Sanya (Evr.1, Sanya city, 18°30′ N 109°52′ E, China) in 2017, Harbin (Evr.2, Harbin city, 45°83′ N 126°84′ E, China) in 2018, and Suihua (Evr.3, Suihua city, 46°63′ N 126°98′ E, China) in 2019. These locations belonged to two soybean ecotopes (Northern and Southern eco-regions) in China, and the soils were Northeast black soil and loam-type soil. Sanya’s climate and soil fertility differed from the other two locations. The irrigation and fertilization regimes of the exact location in 2018 and 2019 were similar.

In addition, the other field management practices were the same as the general practices of the local region. Randomized complete block designs were used for all experiments with three rows, 4 m long and 0.60 m apart, with a space of 6.67 cM between plants. Three replications were performed.

The soybean seeds were matured and dried to a constant weight before analyzing palmitic acid content. Measurement of soybean seeds was conducted using a near-infrared spectrophotometer (NIR) seed analyzer (FOSS NIR DS2500, FOSS Instrument, Denmark). Briefly, approximately 50 g of dry seeds per sample was fitted into a 50 mm diameter cup, and the cup was rotated during NIRS scanning. The average value of three scans per sample was used in data analysis.

### 4.2. Genotype Analysis, Construction, and Genotyping of the SLAF Library, and Construction of the High-Density Genetic Map

The DNA of young, healthy, fresh leaves from both parent lines and all 181 RILs were extracted using the modified CTAB protocol [62]. The specific-locus amplified fragment sequencing library of the two parents and 181 RILs was constructed and sequenced [42]. Based on the soybean reference genome (http://phytozome.jgi.doe.gov/pz/portal.html), we digested genomic DNA using a specific combination of restriction enzymes, RsaI and HaeIII. For construction and genotyping procedures for the detailed library, refer to Ren et al. [40].

### 4.3. QTL Analysis for Palmitic Acid Content

According to the phenotypes of the three environments, QTLs for palmitic acid content were detected using inclusive composite interval mapping (ICIM) in the R/qtl package [63,64,65]. One thousand permutation tests at a 95% confidence level were used to set the LOD threshold. Based on 1000 permutations, LOD = 2.5 was used to determine the presence of a putative QTL associated with a target trait in a particular genomic region.

### 4.4. Validation of Candidate Region and Development of Linkage Marker

In Harbin, 2020, young leaves of 30 plants selected from all population lines were collected and mixed in equal amounts, 30 days after the F10 generation plants were sown at the Nangang experimental station (Harbin, China). Genomic DNA of the two parents and RIL population were extracted by the CTAB method for genotype determination.

Based on the QTL mapping results of SLAF-seq, we screened the polymorphic markers of 27 SSR markers (Table S3) [46] related to the region for verification and linkage markers, which were well-distributed inside and outside of the region. Genomic DNA from parents and 260 RILs were used as templates. The 20-μL PCR contained 100 ng of template DNA, 1.5 mmol/L primers (Tsingke Biotech.), 1 × PCR buffer, 2 mmol/L dNTPs, and 1 unit of Taq polymerase (TransGen Biotech.). The PCR amplification program was as follows: 95 °C for 5 min, 34 cycles of 95 °C denaturations for 30 s, 58 °C annealing for 30 s, 72 °C extensions for 30 s, and a final 72 °C extension for 5 min.

A total of 3 uL 6 × Loading Buffer (loading buffer containing bromophenol blue indicator) was added to a 20 uL sample of PCR amplification product and mixed, then 2 uL of prepared PCR amplification was detected using 6% polyacrylamide gel electrophoresis (PAGE) under 120 V, 400 mA, lasting 30 min. The gel was placed and shaken in 1% AgNO_3_ solution for 3 min and then washed with water; the genotype band was visualized with a developing solution (1000 mL ddH_2_O, 1.5 g NaOH, and 5 mL formaldehyde (35%), mixed). According to general genetic coding rules, the genotypes of the RIL population were assigned as A and B, which were consistent with DN42, Hobbit, and H (heterozygous genotype).

QTL IciMapping was used to validate the candidate region-based SSR marker.

### 4.5. Re-Sequencing and RNA-Seq Analysis of Parents

Re-sequencing and RNA-seq of the two parents were accomplished at Beijing Bio Marker Bioinformatics Technology Co., Ltd. (Beijing, China). For re-sequencing, the DNA of young, healthy, and fresh leaves from both parent lines were extracted using the modified CTAB protocol. Libraries for the four DNA pools were prepared according to Illumina’s library preparation protocol. The nucleic acid sequences of Wm82.a2.v1 (https://www.soybase.org/) was used as the reference genome.

For RNA-seq, developing seeds of the four stages were collected between R5 (beginning seed stage; seed is 1/8 inch long in one of the four uppermost nodes on the main stem; primary and lateral roots grow strongly until R5) and R6 (full seed stage; pods contain a green seed that fills the cavity in one of the four upper most nodes on the main stem; most nutrients have been taken up by the time the plant reaches the R6 stage) [66] from the two parents, DN42 and Hobbit. All tissues were immediately frozen in liquid nitrogen and then stored at −80 °C. Each sample was collected from at least five independent plants and pooled together. According to the supplier’s instructions, total RNA was isolated from the tissues using the RNA prep Pure Plant Kit (TIANGEN, Beijing, China). RNA concentration and quality were measured by spectrophotometer (NanoDrop 2000, Thermo Scientific, waltham, MA, USA). The integrity of RNA was assessed by electrophoresis on a 1% agarose gel and observed by Good View TM staining. First-strand complementary cDNA was synthesized from 2 µg total RNA using the Revert Aid First Strand cDNA Synthesis Kit (Thermo Scientific, Waltham, MA, USA). Each cDNA was diluted 1:10 by adding RNase-free ddH_2_O and stored at −80 °C. qRT-PCR was performed on a CFX96 Touch™ Cycler (Bio-Rad, Hercules, CA, USA) system using SYBR Premix Ex Taq TM II (Takara Biomedical Technology, Beijing, China). Three independent biological replicates were performed for each tissue. Sequencing libraries were generated using NEB Next Ultra^TM^ RNA Library Prep Kit for Illumina (San Diego, USA), following the manufacturer’s recommendations, and sequenced on an Illumina platform. Differential expression analysis of groups was performed using DESeq2. The *p*-values were adjusted using Benjamini and Hochberg’s approach for controlling the false discovery rate. Genes with an adjusted *p*-value < 0.01 found by DESeq2 were assigned as being differentially expressed. RNA-seq analysis was performed using BMK Cloud (www.biocloud.net).

### 4.6. Statistical Analysis

Microsoft Excel 2016 and SPSS19.0 were used for statistical analysis of phenotypic data and correlation analysis. One-way variance (ANOVA) analysis of SSR markers and expression analysis were carried out using the aov function in R.

## 5. Conclusions

In this study, a high-density genetic map related to palmitic acid content was constructed, and two QTLs were mapped based on the genetic map. SSR markers that could be helpful for breeding low palmitic acid content cultivars were developed. Two candidate genes of palmitic acid content were screened.

## Figures and Tables

**Table 1:** Statistical analysis of palmitic acid content (%) in the parent and RIL population over 3 years.

Year	Parents		RILs					
	DN42	Hobbit	Mean	SD	Range	Skewness	Kurtosis	CV (%)
2017	11.99	9.32	9.97	0.8	1.69–11.2	−6.17	61.25	0.08
2018	11.99	8.12	9.52	0.76	1.57–11.03	−6.17	63.94	0.08
2019	10.86	8.92	9.92	0.81	1.9–11.69	−5.25	51.7	0.08

**Table 2:** Characteristics of the high-density genetic map.

Chromosome	Number of Markers	Map Distance (cM)	Average Map Distance (cM)	Gap < 5 cM (%)	Max Gap (cM)
1	539	184.4	0.34	100	4.53
2	170	102.71	0.6	99.41	6.15
3	754	106.6	0.14	100	2.93
4	136	156.16	1.15	100	3.24
5	319	117.18	0.37	99.06	12.36
6	356	137.53	0.39	99.72	5.46
7	401	131.11	0.33	99.75	5.23
8	190	128.56	0.68	100	3.26
9	420	96.8	0.23	100	3.24
10	269	117.89	0.44	98.88	12.47
11	145	116.45	0.8	99.31	5.2
12	134	98.46	0.73	96.99	7.57
13	684	146.24	0.21	99.85	6.49
14	699	112.55	0.16	99.71	8.86
15	414	149.5	0.36	100	4.95
16	845	123.57	0.15	100	2.93
17	969	136.54	0.14	100	4.53
18	1119	169.34	0.15	100	2.43
19	1093	158.84	0.15	99.73	11.84
20	324	112.15	0.35	100	4.53
Total	9980	2602.58	0.39	19.62	5.91

**Table 3:** QTLs of the palmitic acid content of the RIL population in the period 2017–2019.

QTL	Year	Chr.	Left Marker	Right Marker	Genetic Position (cM)		Physical Position (bp)		LOD	ADD	PVE (%)
					Start	End	Start	End			
*qPA10-1*	2017	10	Marker7632755	Marker7723217	76.341	76.341	39,427,048	39,517,256	2.298	0.174	4.151
*qPA15-1*	2018	15	Marker5381863	Marker5394023	43.494	43.78	9,218,139	9,387,049	6.528	0.259	10.066
*qPA15-2*	2019	15	Marker5381863	Marker5394023	43.494	43.78	9,218,139	9,387,049	3.999	0.261	9.526

Chr.: chromosome, LOD: logarithm of odds, PVE: phenotypic variance explained, ADD: additive effect.

**Table 4:** SNPs and Indels in the candidate regions of Dongnong42 and Hobbit.

Gene	SNP							Indel					
	Intergenic	Upstream	Downstream	Nonsynonymous SNV	Synonymous SNV	Intronic	3′UTR	Intergenic	Upstream	Downstream	Non-Frameshift Deletion	Non-Frameshift Insertion	5′ UTR
*Glyma.15g119200*		5	10	2						3			
*Glyma.15g119300*		3	8	1						2	1		
*Glyma.15g119400*		3		1		1	4		3				1
*Glyma.15g119500*		7	6	1			3		4	7		1	3
*Glyma.15g119600*		14	23						2	5			
*Glyma.15g119700*		15	13	2	1		4			3			1
*Glyma.15g119800*		21	20	5					8	3			
Total	319	68	80	12	1	1	11	77	17	23	1	1	5

SNP and Indel numbers were contained in 2000 bp region upstream and downstream from the gene.

## Data Availability

Sequence data from this article can be found in the Phytozome database under the following accession numbers: *Glyma.15g119500*, *Glyma.15g119600*, *Glyma.15g119700*, and *Glyma.15g119800*.

## Supplementary Materials

The following supporting information can be downloaded at: https://www.mdpi.com/article/10.3390/ijms231911273/s1.
