# Supplementary material for: QTL Mapping of Palmitic Acid Content Using Specific-Locus Amplified Fragment Sequencing (SLAF-Seq) Genotyping in Soybeans (Glycine max L.)
Source: Int J Mol Sci. 2022 Sep 24;23(19):11273. doi: 10.3390/ijms231911273 (PMC9569734; doi:10.3390/ijms231911273)
Supplement: Supplementary file 1 [file ijms-23-11273-s001.zip › ijms-1882774-supplementary.pdf]

International Journal of *Molecular Sciences*

MDPI

Article

QTL Mapping of Palmitic acid Content Using Pecific-Locus Amplified Fragment Sequencing (SLAF-Seq) Genotyping in Soybean (*Glycine max* L.)

Table S1. Correlation analysis of 3-year palmitic acid phenotypes.

	2019 Suihua	2018 Harbin	2017 Sanya
2019 Suihua	1		
2018 Harbin	0.986084	1	
2017 Sanya	0.959057	0.970223	1

Table S2. Spearman coefficients of the chromosomes.

Chromosome	Spearman Coefficient	Chromosome	Spearman Coefficient
1	0.9030	11	0.9941
2	0.9994	12	0.9990
3	1.0000	13	1.0000
4	0.9733	14	0.9999
5	0.9999	15	1.0000
6	1.0000	16	0.9999
7	1.0000	17	1.0000
8	0.9979	18	1.0000
9	0.9999	19	1.0000
10	0.9999	20	0.9999

Table S3. 27 SSR markers of chromosome 15 in this research.

Primer ID	Position of Wm82.a2.v1	F: Sequence of Primer (5′–3′)	R: Sequence of Primer (3′–5′)	Size (bp)
G0406	9029987	CCTGTATCTGCTGAGGGTCC	AAAGACAATGAGGGTGCAGG	132
G0407	9064798	GTGCAGCCAAATAACCACAA	CGTAAATGACAAAAACTAAAAATATCA	210
G0408	9074637	TCTGGAAAACTCTTTGTCGCT	TTGACAGCACCGCTATTCAG	247
G0409	9135893	CCACACCCTCTTTGTTGCTT	CATCGGCTACATTTCGGTTT	251
G0410	9149413	AGGCCAACCAATCAACTCAG	TCAGCCATTAAAAATTGGAGAGA	179
G0411	9156119	AAAGCCAGTGTGGCACTTTC	CATGTAGCCGACTCCACCTA	293
G0412	9177704	CCATGCACCTCACTTGGTAA	GCACCTCATTTTGGAGCAGT	186
G0413	9205460	TCTTTGGCAAGTTTGCCTTT	GGGAACCAATCCCCTTTTTA	276
G0414	9219416	CGAGACCAACACAAATTGGA	TGACTTCACTTCCGCTTCCT	236
G0415	9288528	TGATTGAGTAGAGAACGAGAAAAGA	GGAAGTGTTTCTCCTGATTTGG	277
G0416	9330785	CAACCATCCTTTAACCCTGC	TCCATTGACATAAGCCACCA	126
G0417	9338978	TGCTAAAAGAGCGCTAACAAAA	TGGTTGATCTTAAATGAAATCTAAAAA	187
G0418	9350000	AAGGCCATTTTAATAGCTTCATGT	TAGAATTTCTTCGGTTGGCG	242
G0419	9350257	GAAATTAAGCAATACCCCGGA	ACTTTTTGCTCCCGATGAAT	261
G0420	9358076	CAGTTTCAAAAATATAATCAGACATCA	TGTTGTTTGTTCACCTCGTG	123
G0488	9379798	GCAAAGAATGAGAAGCAAGCA	TATGCCCCGTGTGAGTATGA	222
G0487	9396695	GCGTTTCCTAGGGTAAGCACAGTGTATT	GCGGGGTGGAGAGAAAAAGAAAGAACA	248
G0486	9406322	CAATCCCGCCACGTTTATTA	GGTTATCTCATTGTGCAAATTATTG	295
G0485	9412275	ACGGCAAAGCCTAACAAAAA	CAACTCAACGATCCAAAATGAA	281
G0484	9420162	TTACTGAAATCTTGAGAACTGAAAAA	CGCCTACGGATGACTTGC	293
G0482	9441740	TTGGCTAGTCTTCCTTAATTGC	TACCGATTTTGATCCATGCC	268
G0483	9466527	AGGAGCGCTGCTTAGGTTTA	TTCCAGATCTTTTAAGGCCAA	186
G0421	9480647	TGATTTGTGCGTGTGTGTTTT	TCTTTTTATTACACACAAAACGATTG	266
G0422	9497978	CTTCTTCCTCATGCCTCTGC	CTTTTTCCTTGCGATTTTCG	177
G0423	9501396	TCTCTCTTCCCTCCTCCTCC	TCACTTAAAACAAAGTCGTGTGA	222
G0424	9512616	TCGATAGTTAAAGGGCACGG	GCGTGCAATTTCAAGGAGTT	275
G0425	9514715	GGTGTTGCCAAGAATGGACT	AGGGAGGTTAACAGTTGCCA	284

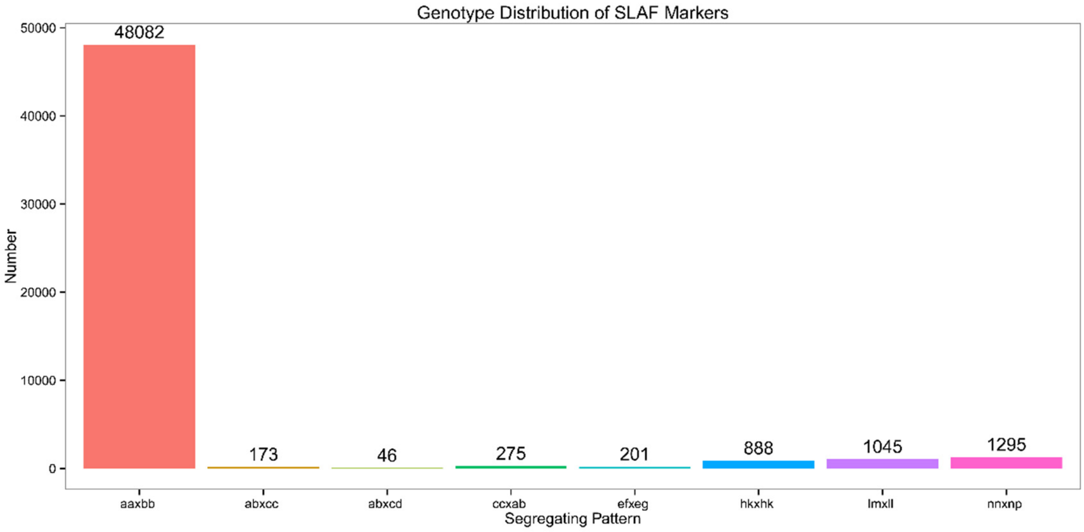

Figure S1. Genotype distribution of SLAF markers.

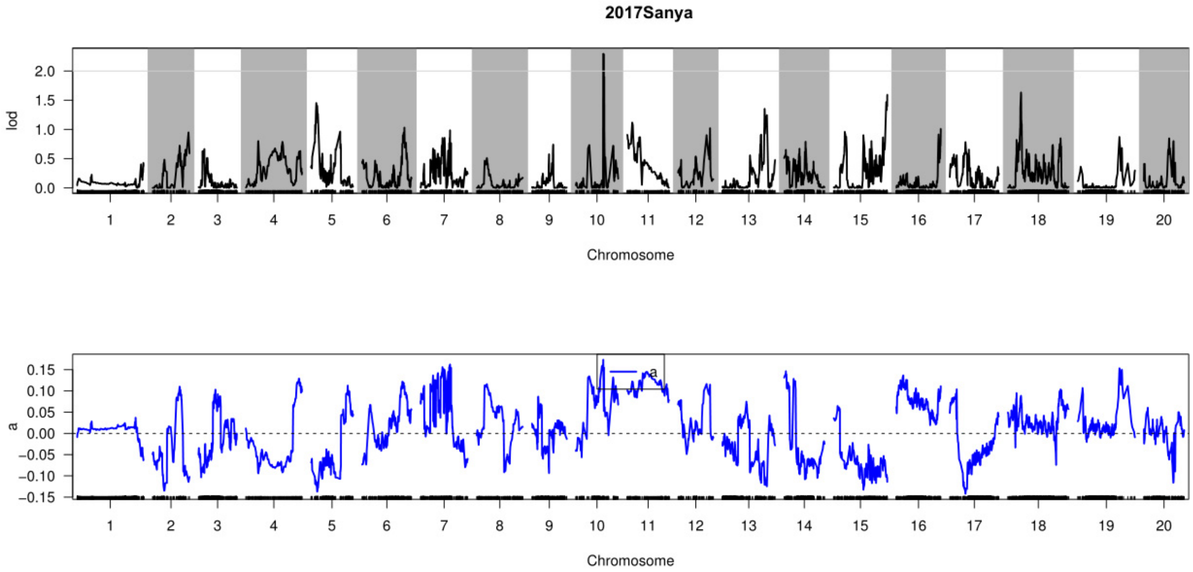

Figure S2. LOD distribution in genome in 2017 Sanya.

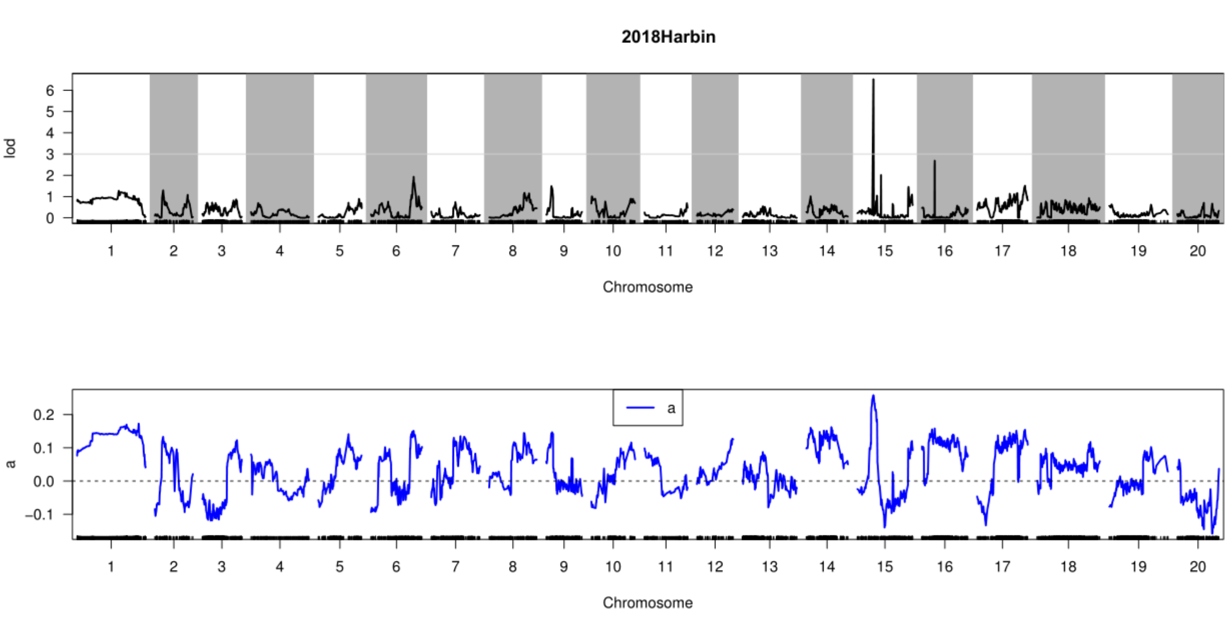

Figure S3. LOD distribution in genome in 2018 Harbin.

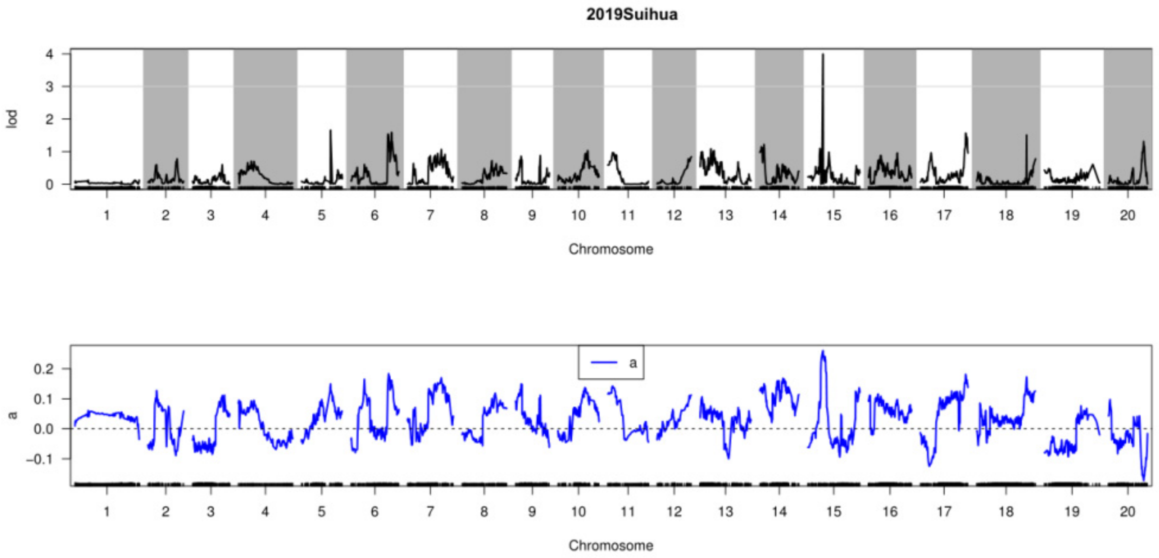

Figure S4. LOD distribution in genome in 2019 Suihua.

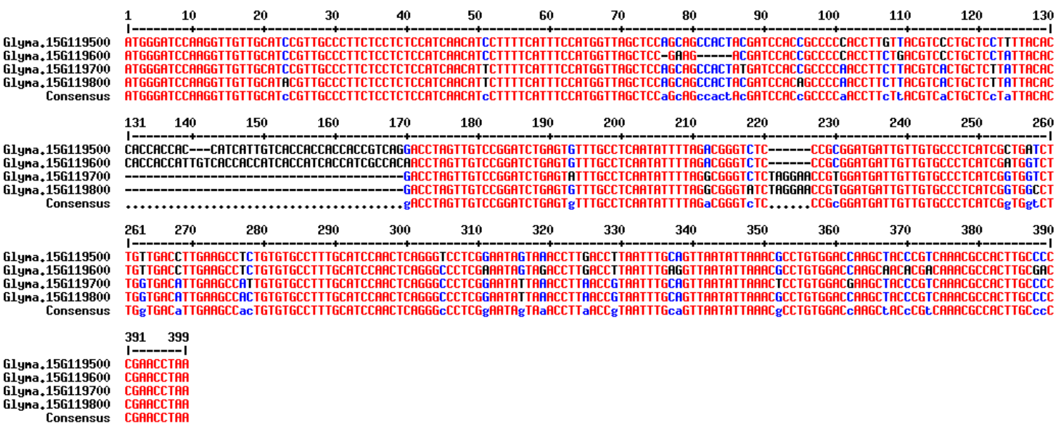

Figure S5. CDS sequence of four genes.

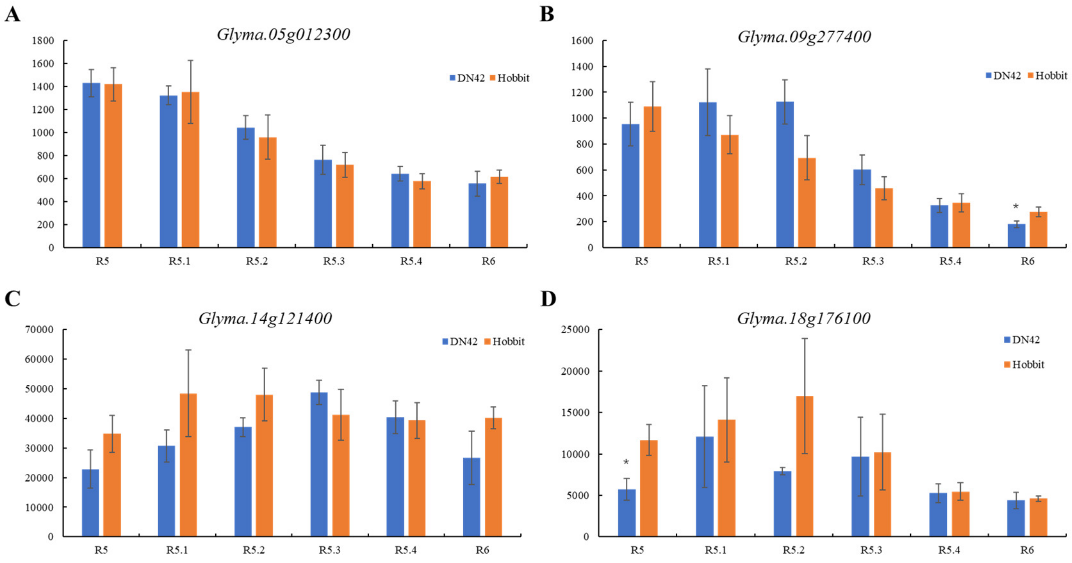

Figure S6. Expression levels of four palmitic acid-related genes (*Glycine max Wm82.a2.v1*) between DN42 and Hobbit.
